# Iatrogenic Acute Type A Aortic Dissection following Elective Cardiopulmonary Bypass

**DOI:** 10.1055/s-0040-1715124

**Published:** 2020-12-23

**Authors:** Pierre Huette, Christophe Beyls, Mathieu Guilbart, Guillaume Haye, Jérémie Vial, Gilles Touati, Yazine Mahjoub, Osama Abou-Arab

**Affiliations:** 1 Department of Anesthesia and Critical Care, Cardiac Thoracic Vascular and Respiratory Intensive Care Unit, Amiens University Medical Center, 1 rue du Professeur Christian Cabrol, Amiens, France; 2 Cardiac surgery Department, Amiens University Medical center, 1 rue du Professeur Christian Cabrol, Amiens, France; 3 Radiologic Department, Amiens University Medical Center, 1 rue du Professeur Christian Cabrol, Amiens, France

**Keywords:** cardiopulmonary bypass, iatrogenic acute aortic dissection, lower limb ischemia

## Abstract

We report a 62-year-old woman who was scheduled for an elective Tirone David valve sparing aortic root replacement under cardiopulmonary bypass. Within the next few hours, the patient developed bilateral acute ischemia of both lower limbs. A thoracic and abdominopelvic computed tomography scan showed acute Type A aortic dissection with a perforation at the brachiocephalic arterial trunk and a complete malperfusion of the inferior mesenteric and iliac arteries.

Iatrogenic acute Type A aortic dissection (AAAD) following cardiopulmonary bypass may occur intraoperatively (with recognition during the primary cardiac surgery), within the first 2 weeks, classified as early postoperative AAAD or late (>30 days after primary heart surgery). 1

We report the case of a 62-year-old woman without previous medical history. After discovery of an aortic murmur, echocardiography demonstrated 3+ aortic insufficiency and an aneurysm of the ascending aorta. Computed tomography (CT) scan revealed an ascending aortic diameter of 70 mm.

The patient underwent an elective David valve sparing aortic root replacement.

Within the next few hours, the patient developed bilateral acute ischemia of both lower limbs and asymmetric blood pressure in the upper limbs. Transthoracic and transesophageal echocardiography revealed a flap in the distal ascending aorta, the aortic arch, and the descending thoracic aorta, suggestive of acute aortic dissection. A thoracic and abdominopelvic CT scan with three-dimensional volume rendering was performed. It showed an acute Type A aortic dissection with fenestration at the brachiocephalic arterial trunk and complete malperfusion of the inferior mesenteric and iliac arteries ( Figs. 1 2 3 ).

**Fig. 1:**
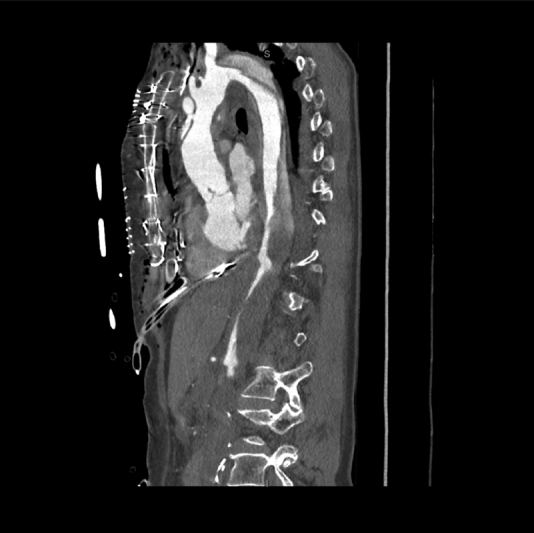
Angio-computed tomography scan on sagittal view showing an acute Type A aortic dissection with complete malperfusion of inferior mesenteric and iliac arteries.

**Fig. 2:**
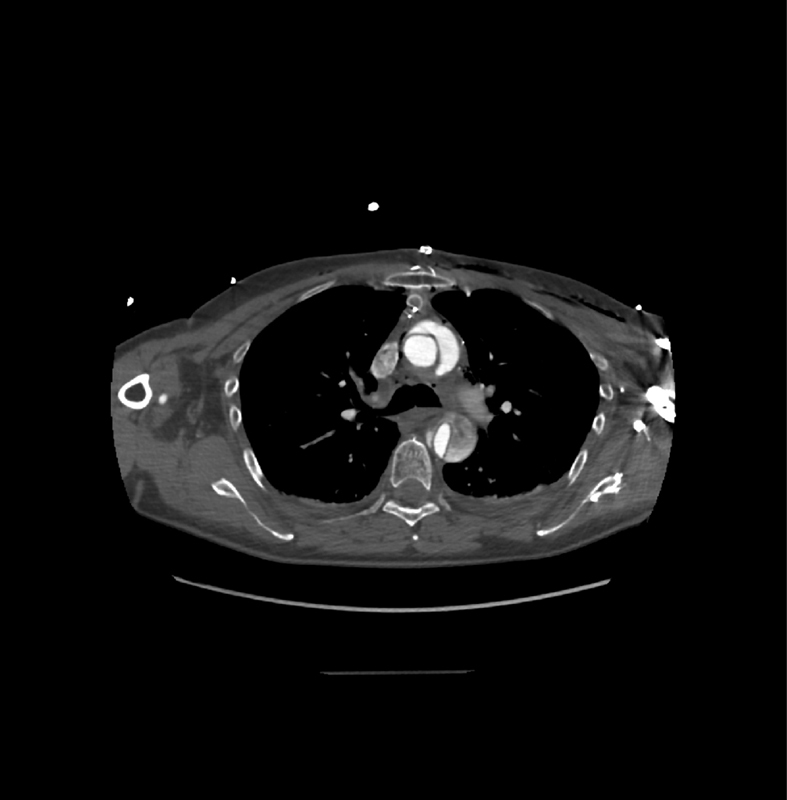
Angio-computed tomography scan on axial view showing an acute Type A aortic dissection with compression of the true lumen.

**Fig. 3:**
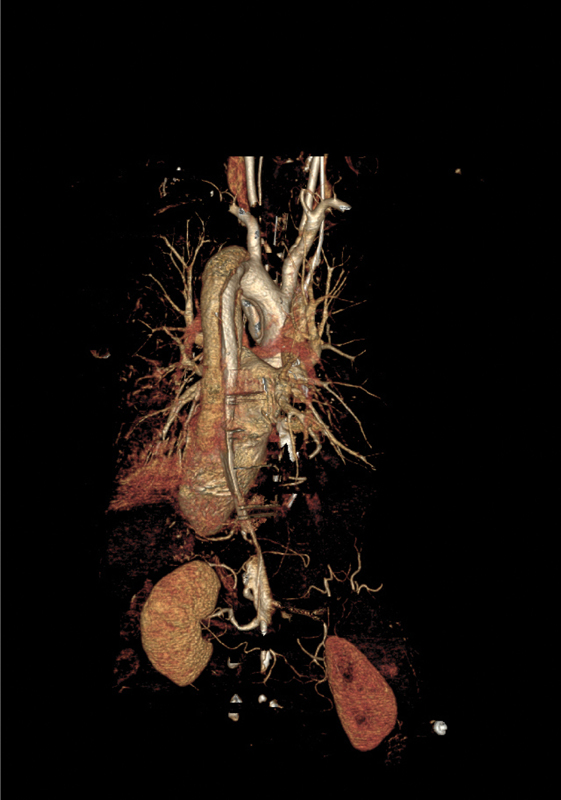
Three-dimensional volume rendering showing acute Type A aortic dissection.

Despite maximal management with revascularization of the lower extremities by axillofemoral bypass, total colectomy, and stenting of the abdominal aorta, evolution was unfavorable with multiorgan failure leading to the death of the patient 72 hours later. Iatrogenic AAAD is a rare complication that must be recognized early to deliver the appropriate therapy. Intraoperative diagnosis may be difficult. 2 3 Regarding the aortic repair, our chosen strategy was a rescue therapy via revascularization of the lower limbs, without direct aortic repair because of the high surgical risk associated with emergency aortic surgery. Shea and Polanco conducted a retrospective study and reported a standardized approach management of AAAD with aortic repair. 4 Thus, in our case, aortic arch repair could have been performed once aortic dissection was recognized. 5 6
